# Local injection of mesenchymal stem cells protects testicular torsion-induced germ cell injury

**DOI:** 10.1186/s13287-015-0079-0

**Published:** 2015-05-30

**Authors:** Chi-Hao Hsiao, Andrea Tung-Qian Ji, Chih-Cheng Chang, Chien-Jui Cheng, Liang-Ming Lee, Jennifer Hui-Chun Ho

**Affiliations:** Department of Urology, Wan Fang Hospital, Taipei Medical University, #111, Section 3, Hsing-Long Road, Taipei, 116 Taiwan; Graduate Institute of Clinical Medicine, Taipei Medical University, #250 Wu-Hsing Street, Taipei, 110 Taiwan; Center for Stem Cell Research, Wan Fang Hospital, Taipei Medical University, #111, Section 3, Hsing-Long Road, Taipei, 116 Taiwan; Department of Pulmonary Medicine, Shuang Ho Hospital, Taipei Medical University, #291, Zhongzheng Road, Zhonghe District, New Taipei, 235 Taiwan; Department of Pathology, College of Medicine, Taipei Medical University, #250, Wu-Hsing Street, Taipei, Taiwan; Department of Pathology, Taipei Medical University Hospital, Taipei Medical University, #250, Wu-Hsing Street, Taipei, 110 Taiwan

## Abstract

**Introduction:**

Testicular torsion is a urological emergency and infertility is a common complication due to ischemic injury. Surgical reduction and orchiopexy is indicated, but to date there is no effective method for restoration of spermatogenesis. The effects of mesenchymal stem cells (MSCs) on acute tissue injury have been demonstrated, and the abilities of paracrine support, differentiation and immune-modulation may benefit to testicular torsion-induced infertility. We investigate the therapeutic efficacy and the mechanisms of MSCs in testicular torsion-induced germ cell injury when injected locally.

**Methods:**

Six to eight-week-old *Sprague–Dawley* rats received surgical 720 degree torsion for 3 hours, followed by detorsion on the left testis. 20 μl of phosphate-buffered saline (PBS) without or with 3 x 10^4^ MSCs from human orbital fat tissues (OFSCs) were given for 10 rats, respectively, via local injection into the left testis 30 minutes before detorsion. 20 μl of PBS injection for 6 rats with surgical exposure without torsion served as sham control. Histopathology with Johnsen’s score analysis, Western blot analysis for superoxide dismutase 2, Bax, Caspase-3, human insulin growth factor-1 and human stem cell factor, malondialdehyde (MDA) assay in testis and plasma, hormones level including testosterone, follicle-stimulating hormone (FSH) and luteinizing hormone (LH) by ELISA Kits, terminal deoxynucleotidyl transferase dUTP nick end labeling (TUNEL) assay and fluorescence staining for P450, Sox-9 and VASA were performed.

**Results:**

Animals were sacrificed and bilateral orchiectomy was performed 7 days after torsion-detorsion. Local injections of OFSCs prevented torsion-induced infertility judging from Johnsen's score. TUNEL assay and Western blot analysis on caspase 3 and Bax demonstrated that OFSCs prevented ischemic/reperfusion induced intrinsic apoptosis. MDA assay revealed that OFSCs significantly reduced the oxidative stress in the damaged testicular tissues. After the OFSC injection, serum testosterone secretion was increased, while the elevation of FSH triggered by testicular injury was balanced. OFSCs also produced stem cell factor in the damaged testis. Immunofluorescence staining revealed that most transplanted cells surrounded the Leydig cells. Some of transplanted cells differentiated into p450 expressing cells within 7 days.

**Conclusions:**

Local injection of allogenic MSCs before surgical detorsion is a simple, clinical friendly procedure to rescue torsion-induced infertility.

## Introduction

Testicular torsion is an emergency among the acute scrotal diseases with the initial presentation of sudden onset, intractable pain due to decreasing blood flow to the testis. The incidence of testicular torsion is around 1/4,000 of the male population younger than 25 years old [1]. There are two prognostic factors of germ cell injury: the duration of testicular ischemia and the severity of cord twisting. It is well accepted that reduction and fixation of the twisted cord within 6 hours significantly reduces the rate of permanent dysfunction on the testis. However, in a high degree of cord twisting, cell necrosis is observed within 4 hours. It is reported that complete or severe testicular atrophy can be found in all patients with cord twisting higher than 360° plus a symptom duration longer than 24 hours [1].

Under testicular torsion and detorsion, ischemic injury accounts for the initial pathomechanism and then reperfusion injury comes next. An ischemia–reperfusion (I/R) injury to the testis not only results in impaired spermatogenesis, but also triggers numerous toxic substances produced by the damaged tissue into the circulation. In addition, vascular endothelial cell injury and induction of microcirculation disorders during reperfusion are harmful to survival of the testis. Production of free radicals such as reactive oxygen species and nitric oxide make a vicious circle of I/R injury [2-4].

In general, the lifetime of mature sperm is 5 to 7 days in the seminiferous tubule [5]. Infertility is a common sequela of torsion-induced ischemia injury followed by testicular necrosis, and impaired spermatogenesis occurs in most of the patients with testicular torsion. Sperm counts less than 20 million/ml can be found in 36% of patients after testicular torsion [6]. In addition to the involved testis, recent studies suggest that damage to the contralateral intact testis is observed, which is caused by antisperm antibody production, altered micro-circulation, and germinal epithelial apoptosis [6]. To date, there is no established standard treatment for testicular torsion-induced infertility.

Theoretically, therapy for ameliorating ischemic injury, promoting spermatogenesis, or regulating immune reaction potentially prevents the complications from testicular torsion. Multipotency and tissue support regulated by the niche environment make stem cells possess the ability for tissue regeneration [7,8]. Among stem cells, mesenchymal stem cells (MSCs) are known to be potent immune modulators [9] and their potential therapeutic benefits on acute, ischemic disorders such as acute myocardial infarction [10], stroke [11], traumatic brain injury [12], and acute liver failure [13] have been reported. Little is currently known about MSCs for acute, ischemic germ cell injury. The therapeutic benefit as well as the underlying mechanism of MSCs on testicular torsion-induced infertility has not been investigated.

Orbital fat-derived stem cells (OFSCs) are MSCs isolated from human orbital fat tissue [14] and their therapeutic effects on acute tissue injury have been demonstrated via paracrine tissue support, immunomodulation, and differentiation ability in our previous experimental studies [15-17]. In this study, rats received surgery with 720° of unilateral testicular torsion for 3 hours, and local injection of OFSCs 30 minutes before surgical detorsion was performed. A standard biopsy testicular score – that is, Johnsen’s score – was used for evaluating spermatogenesis. The reactive oxygen species level and the underlying mechanism of OFSCs in the first 7 days were explored.

## Materials and methods

### Animals

Male Sprague–Dawley rats, 5 to 7 weeks old, were purchased from BioLASCO Taiwan Co., Ltd (Taipei, ROC). The rats were housed at a temperature of 24 ± 3°C and maintained under a 12-hour light–dark cycle. The animals were fed with a standard pellet diet and water *ad libitum*. Rats received surgical torsion–detorsion at the age of 6 to 8 weeks after a 7-day period of acclimatization.

### Isolation and culture of orbital fat-derived stem cells

Isolation and culture of the OFSCs were approved by the Institutional Review Board of Wan Fang Hospital, and were performed as described previously [14]. All samples were removed with the written informed consent of the subjects and followed the regulations of the Institutional Review Board. Briefly, adipose tissues removed from orbital cavity were fragmented, digested, and filtered. After centrifuging the fluid, cells from the resulting pellet were plated in noncoated tissue culture flasks (BD Biosciences, Franklin Lakes, NJ, USA) and maintained in Mesen Pro Medium (Invitrogen, Carlsbad, CA, USA). For the quality control of transplanted cells, characteristics of MSCs including the growth curve, surface phenotyping (positive for MSC markers (CD29, CD90, CD105) and negative for hematopoietic markers (CD31, CD34, CD45, CD106)), and trilineage differentiation capacity of OFSCs had been checked before transplantation. In this study, the cell count was performed with trypan blue staining and the cell viability was 91 ± 2%.

### Experimental protocol

The experimental protocol was approved by the Ethical Committee on Animal Research of Wan Fang Hospital. The rats were randomly allocated into three groups: control group (Ctrl group), six animals received sham operation (surgical incision without testicular torsion); torsion–detorsion group (T/D group), 10 animals received surgery of testicular torsion and detorsion; and torsion–detorsion with OFSC treatment (T/D + OFSC group), 10 animals received surgery of testicular torsion and local injection of OFSCs before detorsion.

All surgical procedures were performed using a sterile technique under anesthesia with intraperitoneal injection of 50 mg/kg ketamine and 45 mg/kg xylazine (Ketalar and Citanest, 2%; Eczacıbas¸ı, Turkey). After left inguinoscrotal incision, unilateral testicular torsion was created by a 720° clockwise rotation on the left testis followed by hemiscrotum fixation with 4–0 atraumatic silk suture for 3 hours. The rats were kept sedated with ketamine and the explored left-side testis was protected with wet gauze and warm light during the 3 hours. At the time of 2.5 hours after torsion, OFSCs were delivered as one shot directly via a needle puncture into the left central testis, and cells were pushed out while moving the needle backward slowly and steadily. Thirty minutes after OFSC trans-plantation, the spermatic cord was detorsed via surgical reduction, and then the wound was closed. All rats were returned to their cages under autoregulating thermal light to maintain body temperature at 37°C after the surgical intervention. After 7 days, bilateral orchiectomy was performed, and blood from the vena cava inferior and tissue samples was obtained.

### Orbital fat-derived stem cell transplantation

In our previous study, the optimal therapeutic dosage of OFSCs for transplantation including acute tissue injury was 3 × 10^7^ cells/kg bodyweight [15,18]. In this study, 3 × 10^4^ cells in 20 μl phosphate-buffered saline (PBS) was chosen as the therapeutic dosage based on the weight of one testis, and local injection of 20 μl PBS served as control.

### Histopathology

Fresh tissues were washed with ice-cold PBS (10 mM Na_2_HPO_4_, 10 mM KH_2_PO_4_, 0.9 g NaCl/100 ml, pH 7.4) and kept at −70°C until assayed. Paraffin-embedded testis were sectioned at a thickness of 5 mm and stained with hematoxylin and eosin. The histopathological score of the testis was evaluated independently by a pathologist using a light microscope. The severity of germ cell injury was qualified by Johnsen’s score, which is also termed the mean testicular biopsy score, from 1 to 10 points as follows: 1 point, no seminiferous epithelium; 2 points, no germinal cells, Sertoli cells only; 3 points, spermatogonia only; 4 points, no spermatozoa or spermatids, few spermatocytes; 5 points, no spermatozoa or spermatids, many spermatocytes; 6 points, no spermatozoa, no late spermatids, few early spermatids; 7 points, no spermatozoa, no late spermatids, many early spermatids; 8 points, less than five spermatozoa per tubule, few late spermatids; 9 points, slightly impaired spermatogenesis, many late spermatids, disorganized epithelium; and 10 points, full spermatogenesis [19]. The value of Johnsen’s score in each testis was the mean point value from at least 10 seminiferous tubules [19].

### Western blot analysis

Testis extracts were lysed, and the proteins purified from the cell lysates were prepared. Western blot analysis were performed using primary antibodies against superoxide dismutase 2 (1:2,000; Abcam, Cambridge, MA, USA), Bax (1:1,000; Santa Cruz, Dallas, TX, USA), Caspase-3 (1:1,000; Cell Signaling, Danvers, MA, USA), human insulin growth factor-1 (IGF-1) (1:1,000; AbCam) or human stem cell factor (SCF) (1:10,000; AbCam), and then secondary antibodies against the fragment crystalizable region of primary antibodies. The density of protein bands was assessed using a computing densitometer with Image-Pro plus software (Media Cybernetics, Inc., Rockville, MD, USA).

### Measurement of oxidative stress level

Lipid peroxidation (malondialdehyde (MDA)) levels in rats’ testis tissue and plasma were detected by MDA Assay Kit (Abcam). Ten milligrams of tissue were homogenized on ice in 300 μl MDA Lysis Buffer (Abcam) and then centrifuged (13,000 × *g*, 10 minutes) to remove insoluble materials. Ten microliters of plasma were mixed with 500 μl of 42 mM H_2_SO_4_ and 125 μl phosphotungstic acid solution at room temperature for 5 minutes. After centrifuging (13,000 × *g*, 3 minutes), the pellet was resuspended on ice with 100 μl double-distilled H_2_O. Then 200 μl solution and 600 μl 2-Thiobarbituric acid solution were incubated at 95°C for 60 minutes, before cooling to room temperature in the ice bath for 10 minutes. The intensity of absorbance at 532 nm was proportioned to the MDA level.

### Measurement of hormone levels

The blood sample was collected via the animals’ tail vein and then centrifuged (945 x g, 10 minutes) to obtain serum. The serum hormone levels were determined by testosterone enzyme-linked immunosorbent assay (ELISA) kit (Abnova, Taipei, Taiwan), follicle-stimulating hormone (FSH) ELISA kit (Abnova), and luteinizing hormone (LH) ELISA kit (Abnova), respectively. The absorbance at 405 nm for testosterone and at 450 nm for FSH and LH was measured.

### Detection of cell apoptosis

To detect the apoptotic cells, the testis sections were stain with the Apo-BrdU-IHCTM In Situ DNA Fragmentation Assay Kit (terminal deoxynucleotidyl transferase dUTP nick end labeling assay; BioVision, Milpitas, CA, USA) and counterstained with methyl green.

### Immunohistochemistry and fluorescence staining

For immunohistochemical staining, tissue sections were incubated with antibodies against superoxide dismutase 2 (Abcam) for 2 hours. The staining was detected using the streptavidin–biotin peroxidase complex method with the DAB Peroxidase Substrate Kit (SK-4100; Vector Laboratories, Burlingame, CA, USA), and counterstained with hematoxylin. For fluorescence staining, frozen-section tissue slides were fixed and blocked, and then slides were triple stained with: mouse antibody against human beta-2-microglobulin (hβ2M; Abcam) followed by DyLight 488-conjugated goat anti-mouse IgG (Jackson ImmunoResearch Laboratories, Sacramento, CA, USA); rabbit antibody against human IgG (Abcam), human/rat sex determining region Y-box 9 (Sox-9; Abcam), or human/rat P450scc (Abcam) followed by DyLight 594-conjugated goat anti-rabbit IgG (Jackson ImmunoResearch Laboratories) at room temperature for 30 minutes; and 4,6-diamidino-2-phenylindole (Santa Cruz) for the nucleus. All samples were assessed under a fluorescence microscope (Leica Microsystem, Wetzlar, Germany). Images were acquired using MetaMorph version 4.6 (Molecular Devices, Sunnyvale, CA, USA).

### Statistical analysis

All values are expressed as the mean ± standard deviation. Analysis of variance was performed for all statistical analyses using a Tukey–Kramer *t* test to perform multiple comparisons between all treatment groups. *P* <0.05 was considered statistically significant.

## Results

### Orbital fat-derived stem cells prevent infertility from testicular torsion

**Figure 1:**
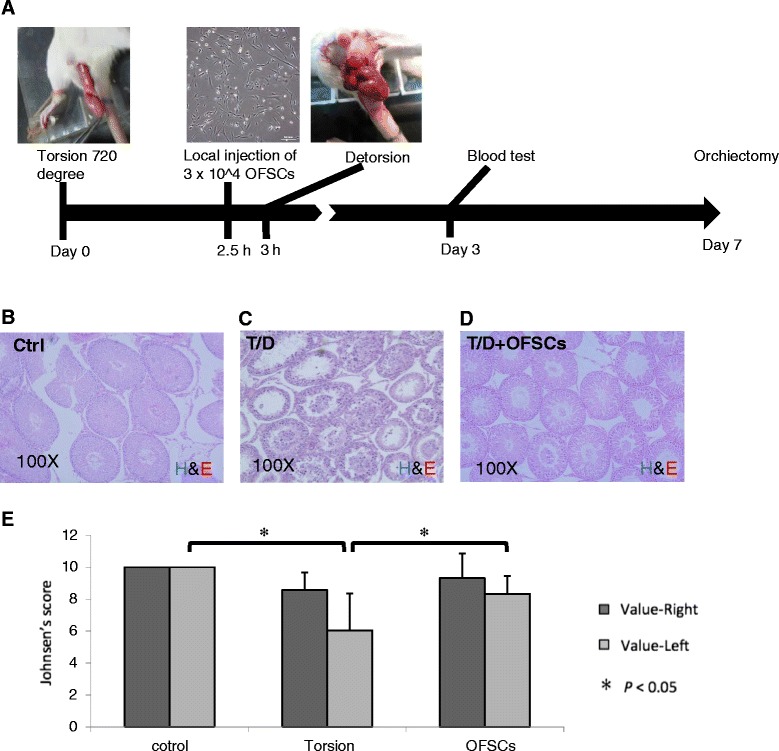
Orbital fat-derived stem cells rescued torsion–detorsion-induced spermatogenic failure. **(A)** Rats received surgical 720° testicular torsion for 3 hours on the left testis, and 3 × 10^4^ human orbital fat stem cells (OFSCs) in 20 μl phosphate-buffered saline was administrated via local injection to the left testis 30 minutes before detorsion. Blood samples were collected on days 0, 3 and 7. Animals were sacrificed for orchiectomy of both testes on day 7. Tissue section of left testis showed **(B)** a normal spermatogenesis in the sham operation group (Ctrl), **(C)** a poor spermatogenesis after torsion–detorsion (T/D), and **(D)** mature sperm formation after T/D with OFSC injection. **(E)** Johnsen’s score demonstrated that OFSCs significantly increased the value of left testis reduced by T/D. H&E, hematoxylin and eosin. Analysis of variance with the Tukey–Kramer *t* test, *P <0.05, *n* = 6.

The study design is illustrated in Figure 1A. Rats were divided into three groups: Ctrl group, T/D group, and T/D + OFSC group. Animals were sacrificed 7 days after testicular torsion and bilateral orchiectomy was performed to obtain all testes (Figure 1A).

The histopathological picture showed a normal spermatogenesis after sham operation (Ctrl group; Figure 1B), and torsion–detorsion (T/D group) resulted in an absence of late spermatids and only few spermatogonia could be found (Figure 1C). In the section of testis with OFSC treatment (T/D + OFSC group), torsion–detorsion-induced damage on spermatogenesis was ameliorated by OFSCs, and mature sperm could be found in some seminiferous tubules (Figure 1D). Using Johnsen’s score to quantify the spermatogenesis on both sides of the testes, we demonstrated that sham operation did not alter the value of Johnson’s score (left value of Ctrl group 10 ± 0 vs. right value of Ctrl group 10 ± 0). Torsion–detorsion significantly reduced the Johnsen’s score (left value of T/D group 6.04 ± 0.62 vs. left value of Ctrl group 10 ± 0), and OFSCs significantly increased the value of Johnsen’s score in a testis with torsion–detorsion (left value of T/D + OFSC group 8.33 ± 1.13 vs. left value of T/D group 6.04 ± 0.62) (Figure 1E). There was no statistical difference with regards to the right testis among the three groups.

### Orbital fat-derived stem cells protect testis from torsion-induced apoptosis

**Figure 2:**
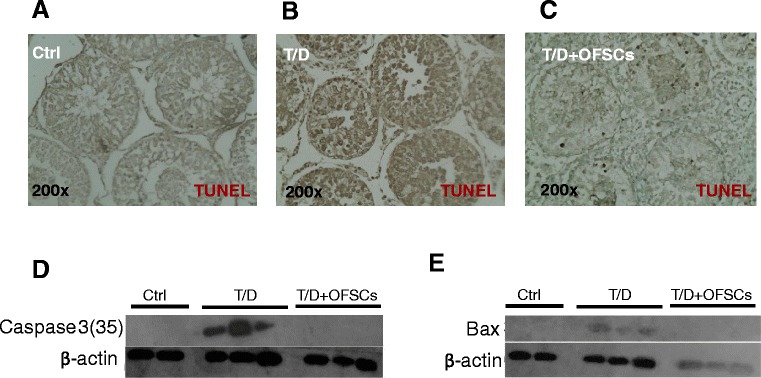
Orbital fat-derived stem cells prevented the damaged testis from apoptosis. **(A)** Testis in the sham operation group (Ctrl) was negative for DNA fragmentation. **(B)** Torsion–detorsion (T/D) induced massive apoptotic bodies which were positive for terminal deoxynucleotidyl transferase dUTP nick end labeling (TUNEL) stain in the cell nucleus located at the seminiferous tubule. **(C)** Only few apoptotic bodies could be seen in damaged testis with orbital fat-derived stem cell (OFSC) injection. **(D)** OFSCs abrogated the testicular torsion-induced caspase 3 expression. **(E)** The protein levels of bax were undetectable in the sham operation group (Ctrl) and T/D with OFSCs.

To determine whether OFSCs restored the tissue death from torsion-induced testicular injury, terminal deoxynucleotidyl transferase dUTP nick end labeling assay was applied on the tissue section. As shown in Figure 2A,B,C, testis in the sham operation group was negative for DNA fragmentation (Figure 2A), while torsion–detorsion induced massive apoptotic bodies in the cell nucleus located at seminiferous tubules (Figure 2B). However, OFSCs significantly reduced the apoptotic bodies triggered by testicular torsion injury (Figure 2C). Western blot analysis revealed that OFSCs abrogated the testicular torsion-induced caspase 3 (Figure 2D) and bax (Figure 2E) expression in the testis.

### Orbital fat-derived stem cells reduce torsion-induced oxidative stress in the testis

**Figure 3:**
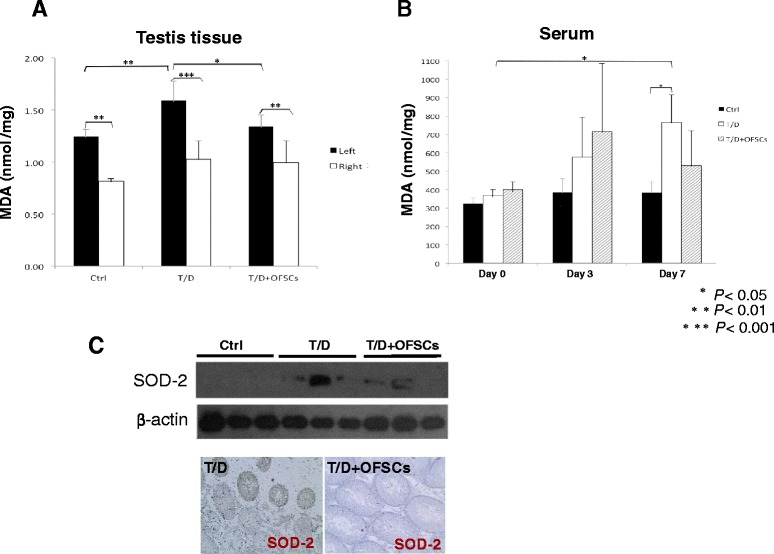
Orbital fat-derived stem cells ameliorated local oxidative stress elevated by torsion–detorsion. **(A)** Sham operation did increase the malondialdehyde (MDA) level in the left testis. Torsion–detorsion (T/D) significantly increased oxidative stress in the left testis, and orbital fat-derived stem cells **(**OFSCs) effectively reduced the MDA level elevated by T/D. **(B)** Oxidative stress in the circulation progressively increased by T/D, but OFSCs did not significantly alter the circulating MDA level in the first 7 days. Analysis of variance with the Tukey–Kramer *t* test, *P <0.05, **P <0.01, ***P <0.001, *n* = 6. **(C)** Production of superoxide dismutase 2 (SOD-2) in testis tissue was induced by T/D, and OFSC injection subsided the response.

Bax expression results in leakage of cytochrome c from mitochondria which activates the intrinsic pathway of apoptosis in germ cells [20]. It has already been established that torsion–detorsion increased the oxidative stress in testis which triggers apoptosis from the intrinsic pathway [2-4]. We further measured the oxidative stress by lipid peroxidation assay. We demonstrated that sham operation, torsion–detorsion or OFSCs on torsion–detorsion did increase the oxidative stress in the testis (Figure 3A, left vs. right in Ctrl group, T/D group or T/D + OFSC group). Torsion–detorsion significantly upregulated the oxidative stress in the damaged testis in comparison with sham operation (Figure 3A, left side MDA in Ctrl group vs. left side MDA in T/D group), and OFSCs significantly reduced the elevation of MDA level triggered by torsion–detorsion in the testicular tissues (Figure 3A, left side MDA in T/D + OFSC group vs. left side MDA in T/D group). Oxidative stress in the circulation progressively increased after torsion–detorsion (Figure 3B, white bar day 0 vs. white bar day 7; white bar day 7 vs. black bar day 7), but OFSCs did not significantly alter the circulating MDA level in the first 7 days (Figure 3B, gray bar vs. black bar at each time point). Western blot analysis and immunohistochemical staining demonstrated that superoxide dismutase 2, an anti-oxidative enzyme response to the oxidative stress in mitochondria [21], was produced in testis tissue in response to testicular torsion, which subsided with OFSC injection (Figure 3C).

### Orbital fat-derived stem cells maintain homeostasis of testosterone

**Figure 4:**
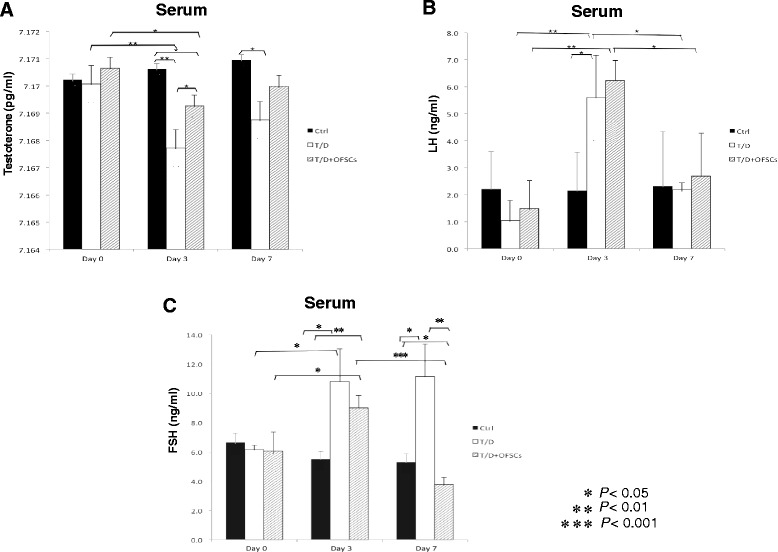
Orbital fat-derived stem cells maintained homeostasis of testosterone. **(A)** Torsion–detorsion (T/D) resulted in reducing serum testosterone, and orbital fat-derived stem cells (OFSCs) progressively recovered the testosterone level in the first 7 days. **(B)** T/D transiently increased the level of luteinizing hormone (LH) on day 3, but OFSCs did not regulate LH in the first 7 days. **(C)** The follicle-stimulating hormone (FSH) level was triggered by T/D testicular injury on day 3 and day 7, and OFSCs returned the FSH level back to normal status on day 7. *P <0.05, **P <0.01, ***P <0.001.

Reproduction relies on the homeostasis of sex hormones. Testosterone is secreted by Leydig cells, stimulating the maturation of spermatids and the elevation of FSH and LH produced by the anterior pituitary gland in the absence of normal restricting feedback from the gonad [22]. ELISA showed that torsion–detorsion resulted in the reduction of serum testosterone level on day 3 and day 7, and OFSCs significantly rescued the torsion–detorsion-induced low testosterone on day 3, and the testosterone level on day 7 returned to the normal status after OFSC injection (Figure 4A). Torsion–detorsion transiently increased the level of LH on day 3, but not on day 7 (Figure 4B, value of white bar at each time point; value of white bar at day 3 vs. value of black bar at day 3). OFSCs did not regulate torsion–detorsion-induced LH changes in the first 7 days (Figure 4B, value of white bar vs. value of grey bar at each time point). Torsion–detorsion upregulated the serum FSH level on day 3 and day 7 (Figure 4C, value of black bar vs. value of white bar at each time point), and OFSCs balanced the FSH level on day 7 (Figure 4C, value of white bar vs. value of grey bar at each time point). However, human testosterone, LH, and FSH were undetectable in this study (data not shown).

### Orbital fat-derived stem cells secret stem cell factor in the testis with torsion–detorsion

**Figure 5:**
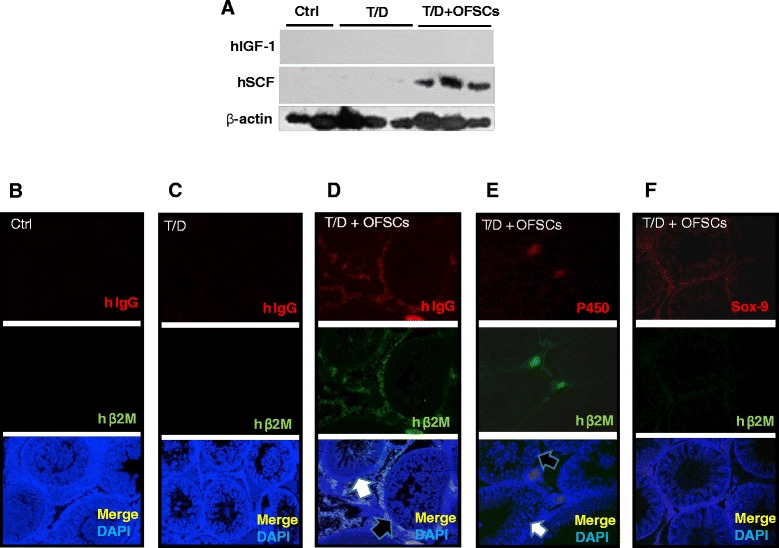
Orbital fat-derived stem cells secreted stem cell factor and supported Leydig cells. **(A)** After orbital fat-derived stem cell (OFSC) injection, stem cell factor (SCF) was abundant in damaged testicular tissue. Human (h) immunoglobulin (IgG) and beta-2 microglobulin (β2M), two probes for human cells, were not detectable in **(B)** the sham operation group (Ctrl) and **(C)** the torsion–detorsion group (T/D). **(D)** Most hIgG-expressing and hβ2M-expressing cells could be detectable in the space between the seminiferous tubules. **(E)** Some of the human cells differentiated into P450-expressing cells and **(F)** very few human cells differentiated into sex determining region Y-box 9 (Sox-9)-positive cells. DAPI, 4,6-diamidino-2-phenylindole.

To explore the key paracrine regulator produced by OFSCs in the damaged testis, western blot analysis was performed to measure the expression of IGF-1 and SCF, two critical secreted factors for supporting spermatogenesis and testosterone secretion [23-26]. As shown in Figure 5A, human IGF-1 and human SCF were undetectable in testis without OFSC injection. The amount of human SCF, rather than human IGF-1, was abundant in damaged testis after OFSC injection.

### Local injection of orbital fat-derived stem cells supports Leydig cells in the testis

To determine the fate and biodistribution of the transplanted cells, two human specific proteins (hβ2M and hIgG) were used for probes of OFSCs by immunofluorescence staining. hIgG and hB2M were not detectable in both the Ctrl group (Figure 5B) and the T/D group (Figure 5C). In a damaged testicular tissue, most human cells could be found in the space between the seminiferous tubules rather than clustered at the central testis (Figure 5D). OFSCs randomly supported spermatogenesis. Some seminiferous tubules near OFSCs showed mature spermatogenesis (Figure 5D,E, white arrow), and some did not (Figure 5D,E, black arrow). Antibodies against human/rat P450, Sox-9, and VASA were used to identify Leydig cells [27], Sertoli cells [28], and sperm [29], respectively. In all 10 testis sections stained with P450, some hB2M-expressing cells differentiated into P450-expressing cells could be observed in each tissue section (Figure 5E). In six of the 10 testis sections stained with Sox-9, only a few of transplanted cells expressed Sox-9 (Figure 5F) in the first 7 days. Among the nine testis sections stained with VASA, no VASA-expressing human cells could be found (data not shown). In the contralateral testis, neither hβ2M-expressing nor hIgG-expressing cells could be identified (data not shown).

## Discussion

In this study we explore whether local injection of allogenic MSCs during surgical reduction for torsion-induced testicular injury is clinically applicable to prevent infertility. The therapeutic benefit of MSCs comes from prevention of testicular apoptosis (Figure 2), reduction of intra-testicular oxidative stress (Figure 3), and promotion of testosterone secretion (Figure 4A), which maintains the spermatogenesis against torsion-induced germ cell injury (Figure 1). Most transplanted cells surround the Leydig cells and secrete stem cell factor for supporting spermatogenesis (Figure 5A), while some of them potentially differentiate into Leydig cells (Figure 5E).

In the clinic, severe germ cell injury longer than 24 hours leads to a persistent infertility [1], and Johnsen’s score is used to evaluate pathological infertility. According to the definition of Johnsen’s score, 10 points represent a full spermatogenesis, 9 points slightly impaired spermatogenesis with many late spermatids and disorganized epithelium, and 8 points less than five spermatozoa per tubule and few late spermatids [19]. Therefore, the mature spermatid (that is, spermatozoa) only exists in the seminiferous tubule if the Johnsen’s score is 8 points or higher, implying that infertility occurs when the Johnsen’s score is below 8 points. In this study, the mean value of Johnsen’s score was less than 6 points after torsion–detorsion. OFSC treatment resulted in the mean value of Johnsen’s score recovery being higher than 8 points (Figure 1E), demonstrating that OFSCs rescue torsion-induced infertility.

In a review of the related literature, there have been a number of reports regarding MSC transplantation protecting kidney [30], heart [31], intestine [32], and lung [33] tissues against I/R-induced injury in experimental models via anti-inflammation, anti-reactive oxygen species, and anti-apoptosis. Testis atrophy is frequently found after testicular torsion; however, the blood–testis barrier physiologically isolates most immune cells out of the testicular tissue and limits the inflammatory response during testicular torsion-induced I/R injury [34]. In this study, tumor necrosis factor alpha, interferon gamma, interleukins, and immune cell markers in the testis were not altered by either torsion–detorsion or OFSCs (data not shown), indicating that anti-inflammation of MSCs is not the dominant mechanism for torsion-induced testicular I/R injury. Except for inflammation inhibition, OFSCs demonstrated a significant effect on ameliorating local oxidative stress and apoptotic prevention after testicular torsion (Figures 2 and 3).

Full spermatogenesis relies on support by Leydig cells and Sertoli cells. Leydig cells are interstitial cells located at interstitial spaces adjacent to the seminiferous tubules. They are testosterone-producing cells in the presence of LH [35]. Testosterone and FSH are essential for spermatogenesis and promote differentiation of spermatogonia via activating Sertoli cells [35]. Also in the related literature, *in vivo* differentiation into Leydig cells from bone marrow-derived MSCs has been described [36]. MSCs were thought to be an effective source of stem cells for producing steroidogenic cells. However, the differentiation potential of MSCs into glucocorticoid-producing cells was higher than that into testosterone-producing cells [36]. There was no evidence which showed that MSCs differentiated into Sertoli cells in the related literature. In addition, MSCs derived from bone marrow demonstrated a better differential ability into germ cells *in vitro* than MSCs from subcutaneous fat tissue [37].

Using the testicular torsion-induced ischemic model, we proved the concept that OFSCs – MSCs derived from human orbital fat tissue – possess *in vivo* differentiation potential into Leydig cells, but are doubtful for Sertoli cells (Figure 5). According to our data, no VASA-positive cells (mature sperm) could be co-stained by human probes (hIgG or hβ2M; data not shown), suggesting that the mature sperm shown in Figure 1D is differentiated from mouse spermatozoa. For allogenic transplantation, MSCs from adipose tissue have a low risk of genetic hybridization into the filial generation. In this study, most transplanted cells surround the Leydig cells, and some of them differentiate into p450-positive cells. The number of differentiation cells in the first 7 days is extremely low compared with the cell number of transplantation (Figure 5E). In addition, human testosterone was undetectable (data not shown) during the first 7 days, suggesting that MSCs do not differentiate into functional Leydig cells within 7 days and paracrine support of the Leydig cells serves as the major mechanism for MSCs.

SCF, also known as kit ligand or steel factor, is a cytokine that binds to the c-Kit receptor. SCF plays an important role in blood cell differentiation, mammalian spermatogenesis, and melanogenesis. c-Kit on the membrane of primordial germ cells, spermatogonia, and in primordial oocytes delivers the signals from SCF, and therefore SCF is essential for the maintenance of primordial germ cells in both sexes [25]. The SCF/c-Kit signaling pathway, like melanoblasts, helps to guide the cell location during development [26]. Besides, germ cell proliferation, cell migration, cell adhesion, and anti-apoptotic actions in testis also involve the SCF/c-Kit [38]. In our current study, human SCF was strongly expressed in damaged testis after OFSC injection, indicating that OFSCs produce SCF to support germ cell proliferation and migration and protect against apoptosis. The involvement of SCF resulted in better spermatogenesis after torsion–detorsion.

According to our data, neither surgical torsion–detorsion (T/D group) nor torsion–detorsion with OFSC injection (T/D + OFSC group) significantly altered the value of Johnsen’s score on the right-side (nontorsion) testis (Figure 1E). Besides, no transplanted cells could be found in the right-side (nontorsion) testis when we performed the fluorescence staining on right-side testis (data not shown). We conclude that local injection of OFSCs into damaged testis has no effect on contralateral, nontorsion testis in the first 7 days.

Most valuable for stem cell transplantation in this study is providing a therapeutic strategy for the urologist. The reason we choose local injection instead of using intravenous injection is that the physiological blood–testis barrier stops stem cells from entering testis tissue via the circulation. Intravenous injection is not a good treatment option in this model from a clinical point of view. In clinical practice, testicular torsion occurs incidentally. Intractable pain makes the patient seek help in the emergency room. According to the clinical standard operation procedure, scrotal exploration and testicular detorsion will be arranged emergently if testicular torsion is suspected [1]. However, detorsion-induced I/R injury will superimpose the torsion-induced ischemic change on the testis [2-4]. To prevent further I/R injury, we prefer pretreatment with allogenic MSCs in the emergency room before surgical detorsion rather than after surgical detorsion. The timing of treatment in this study (that is, 30 minutes before surgical detorsion) is clinical applicable since the preoperation preparation routinely takes 30 minutes for diagnosis by the urologist, anesthesia consultation, and patient transfer. The therapeutic effect of allogenic MSC injection after surgical detection should be further determined.

However, there are a number of limitations regarding this study that need to be taken into consideration. With a 7-day observation it is impossible to evaluate the effect of OFSCs on anti-sperm antibody-induced secondary infertility. Besides, the long-term effect of OFSCs on prevention of infertility and the long-term fate of OFSCs cannot be concluded in this study. Although there is not a significant change from Johnsen’s score for the right (nontorsion) testis after torsion injury (Figure 1E), the impact of OFSCs on the nontorsion testis needs further investigation.

## Conclusion

Seven days after torsion–detorsion there is no obvious injury noted in the right-side testis. MSCs from adipose tissue protect germ cells from testicular torsion-induced infertility mainly through reducing oxidative stress, preventing testis apoptosis and supporting spermatogenesis with SCF secretion. Local injection of allogenic MSCs from adipose tissue before the surgical detorsion provides a new therapeutic strategy to rescue infertility, a sequel of testicular torsion-induced germ cell injury.

## Box 1. About Jennifer Ho

JHH, an Ophthalmologist, is the Associate Professor of Graduate Institute of Clinical Science at Taipei Medical University. Currently, she also serves as the Director of Medical Research Department and Clinical Trial Center for medical research and clinical trial management at Wan Fang Medical Center. She received her Medical Degree from National Taiwan University and PhD in Pharmaceutical Science from National Yang-Ming University. The theme of her research is to optimize the therapeutic efficacy of mesenchymal stem cell (MSC) transplantation. Using diseased animal models, the translational research of MSC transplantation on spinocerebellar ataxia, type 1 and 2 diabetes, acute lung injury, alkali-induced corneal-limbal deficiency and torsion-induced infertility were performed in her lab. In addition, she is interested in modification of biophysical effects in MSCs via photo-irradiation, shear stress, and F-actin cytoskeleton organization to enhance the MSC activities. Her pre-clinical research achievements have supported several MSC clinical trials in Taiwan.

## Note

 This article is part of an ‘Emerging Investigators’ collection showcasing the work of early career investigators who have demonstrated growing leadership in the field of stem cells and regenerative medicine. Other articles in the series can be found online at http://stemcellres.com/series/emerginginvestigators

## Abbreviations

ELISA: enzyme-linked immunosorbent assay
FSH: follicle-stimulating hormone
IGF-1: insulin growth factor-1
hβ2M: human beta-2-microglobulin
I/R: ischemia–reperfusion
LH: luteinizing hormone
MDA: malondialdehyde
MSC: mesenchymal stem cell
OFSC: orbital fat-derived stem cell
PBS: phosphate-buffered saline
SCF: stem cell factor
Sox9: sex determining region Y-box 9
